# The influence of adsorption geometry on the reduction affinity of nitroaromatics on Au(111)†

**DOI:** 10.1039/d2cp02832h

**Published:** 2022-09-12

**Authors:** Iris Berg, Helen Eisenberg, Shahar Dery, Tehila Shahar, Albano Cossaro, Alberto Verdini, Luca Floreano, Tamar Stein, Elad Gross

**Affiliations:** Institute of Chemistry, The Hebrew University Jerusalem 91904 Israel tamar.stein@mail.huji.ac.il elad.gross@mail.huji.ac.il; The Center for Nanoscience and Nanotechnology, The Hebrew University Jerusalem 91904 Israel; The Fritz Haber Center for Molecular Dynamics Research, The Hebrew University Jerusalem 91904 Israel; CNR-IOM, Laboratorio Nazionale TASC Basovizza SS-14 Trieste 34012 Italy

## Abstract

Chemoselective reduction of nitro groups in multifunctional nitroaromatics is a challenging catalytic process with high interest due to the importance of the resulting anilines for the chemical industry. Molecular-level understanding of the ways by which adsorption geometry of nitroaromatics influence their affinity toward nitro reduction will enable the development of highly selective reactions. Herein, taking advantage of the well-ordered self-assembly of *para*- and *ortho*-nitrothiophenol (*p*-NTP and *o*-NTP, respectively) monolayers on Au(111), we examined the correlation between adsorption geometry and nitro reduction affinity. The anchoring geometry of NTPs and their nitro reduction affinity were determined by conducting polarized X-ray absorption spectroscopy while the influence of NTPs′ adsorption geometry on the interaction with the Au surface was analyzed by density functional theory (DFT) calculations. Exposure of surface anchored *p*-NTPs to reducing conditions led to their reorientation from a tilt angle of 52° to 25°, which enabled strong interactions between the π system of the molecules and the Au surface. Direct correlation was identified between the surface proximity of the nitro group, its parallel position to the surface and the resulting reduction yield. The asymmetric structure of *o*-NTP led to a tilted adsorption geometry in which the nitro group was rotated away from the plane of the aromatic ring and therefore was positioned parallel and in high proximity to the Au surface. This positioning led to surface-bonding that involved the oxygen atoms of *o*-NTP. The higher surface proximity and stronger surface interactions of the nitro group in *o*-NTP enabled nitro reduction already at 180 °C, while in *p*-NTP nitro reduction was achieved only at 230 °C, due to the longer distance between the NO_2_ group and the Au surface that led to weaker adsorbate-surface interactions. Thus, parallel positioning of the nitro group and high surface proximity were found as essential descriptors for nitro reduction affinity in both *p*-NTP and *o*-NTP on the Au surface. These findings provide explicit guidelines for tuning the reactant and surface properties in order to control the reactant's adsorption geometry for selective nitro reduction in multifunctional nitroaromatics.

## Abstract 2

The dominant influence of adsorption geometry on chemical reactivity was identified for nitro reduction on Au(111). Reactivity was facilitated only when the nitro group was oriented in high proximity and approximately parallel to the metal surface.

## Graphical abstract

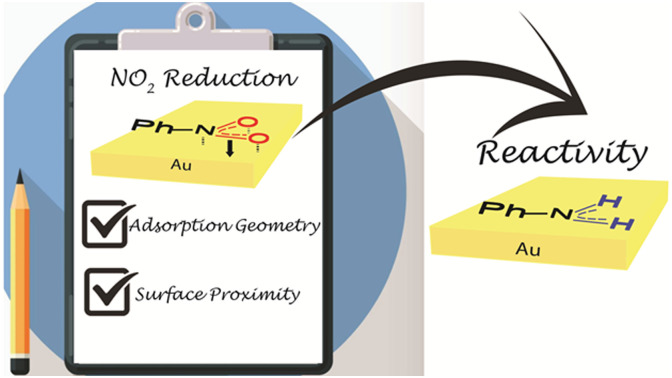

## Introduction

The catalytic reduction of nitroaromatics has spurred interest for decades due to the fact that primary amines, which are the reduction products, are central precursors in the synthesis of pharmaceuticals, polymers and agrochemicals.^1–3^ One of the prominent challenges in this reaction is the selective reduction of nitro groups in the presence of other reducible groups.^4^ This challenge was addressed by the development and utilization of various catalysts.^5–7^ Some of the most promising heterogeneous catalysts for selective nitroaromatics reduction are oxide-supported Au, Pt and Pd nanoparticles.^8–12^ Pt-based catalysts demonstrated high reactivity toward nitro reduction, but the high reactivity was coupled with deteriorated selectivity.^4^ Au-based catalysts, on the other hand, offer a higher degree of selectivity but are characterized with low conversion rate.^13–16^

One of the crucial factors that governs the selectivity toward nitro reduction is the adsorption geometry of multifunctionalized nitroaromatics on catalytic surfaces.^17–20^ The reactants’ adsorption geometry dictates the surface proximity of chemically active groups, influences their affinity toward activation by surface-induced reactions and directs the chemical selectivity.^17,18,20–29^ Thus, classifying the adsorption geometry of nitroaromatics and the ways by which it influences the affinity toward nitro reduction, which is the aim of this work, will enable the design of highly selective catalysts by fine-tuning the surface and/or reactants' properties.

The central influence of adsorption geometry on the reactivity and the ways by which it can be tuned for optimized selectivity was demonstrated in hydrogenation of multifunctional ketones. It was experimentally identified that parallel positioning of the C=O functional group with respect to the catalytic surface is essential for C=O reduction.^26,30–32^ The parallel positioning of the C=O group enabled interaction of both carbon and oxygen atoms with the catalytic surface *via* π bonding. This adsorption geometry induced carbonyl activation through weakening of the C=O bond. Adsorption configuration in which the carbonyl group was positioned perpendicular to the surface did not effectively facilitate the hydrogenation reaction since only the oxygen atom formed a σ bond with the catalytic surface.^32,33^

The potential influence of adsorption geometry on nitro reduction affinity was predicted by density functional theory (DFT) calculations, which identified that vertical adsorption of nitro groups on a Ni surface will induce high selectivity toward nitro reduction,^19^ and that the kinetic barrier in nitrobenezene reduction can be moderated following adsorption of the phenyl group on Pt(111) and Au(111) surfaces.^34,35^ However, experimental validation for the predicted correlation between adsorption geometry and chemical selectivity toward nitro reduction in nitroaromatics has not yet been demonstrated.

It should be noted that direct experimental analysis of the influence of adsorption geometry on the chemical reactivity is highly challenging since it requires a controlled environment in which the adsorption geometry of reactants can be tuned and its influence on the transformation of reactants into products can be monitored. One approach for tuning the adsorption geometry of reactants was based on forming a steric hindrance by using inert self-assembled molecules that altered the reactants’ adsorption geometry from flat-lying to standing position.^36–40^ A different and less restrictive approach was based on comparison of several structural isomers and analyzing the influence of surface proximity on the reactivity.^41^

Herein, the correlation between adsorption geometry, surface proximity and nitro reducibility in nitroaromatics was unveiled by using nitro-functionalized self-assembled monolayers (SAMs) of two isomers, *ortho*- and *para*-nitrothiophenol (*o*-NTP and *p*-NTP, respectively). The NTPs were deposited under Ultra-High Vacuum (UHV) conditions on Au(111) single crystal. By using tethered molecules as a model system, we monitored the influence of adsorption geometry on the reactivity. Such observation cannot be easily achieved with non-tethered molecules due to the strong affinity of the benzene ring to the surface that leads to a flat lying position.^34,35^ The NTPs' adsorption geometry and nitro reduction affinity were probed by conducting polarized near-edge X-ray absorption fine structure (NEXAFS) and X-ray photoelectron spectroscopy (XPS) measurements following exposure to various reducing conditions.^42^ The experimental results indicated that nitro reduction was facilitated when the nitro group was oriented in a close to parallel position and in high proximity to the Au surface. DFT calculations demonstrated that high proximity and close to parallel orientation enabled stronger binding of the nitro group to the Au surface due to orbital overlap between the NTP molecules and the Au surface.

## Methods

*ortho*-Nitrothiophenol (*o*-NTP) (96%) was purchased from Sigma-Aldrich and was used without further purification. *para*-Nitrothiophenol (*p*-NTP) (96%) was purchased from Alfa-Aesar and was used without further purification. Au(111) single crystals with a surface area of 0.5 cm^2^ (purchased from SPL) was cleaned under ultrahigh vacuum conditions by three consecutive cycles of sputtering (2 × 10^−6^ torr Ar; 1.5 keV; 20 min) and subsequent annealing to 500 °C. The cleaning process was validated by monitoring the C1s XPS signal (Fig. S1, ESI†).

Vapor-deposition of *o*-NTP and *p*-NTP was performed in a UHV chamber at ALOISA beamline. The molecules were housed in a home-built evaporator, equipped with a shutter. The nozzle of the evaporator was aimed toward the Au(111) surface. The evaporation temperature was raised to 65 °C and the molecules were dosed into the UHV chamber, inducing an increase in the back-pressure to 2 × 10^−7^ torr. The Au surface was kept at room temperature and exposed to the evaporated molecules for 20 minutes.

Synchrotron radiation XPS measurements were obtained using a *p*-polarized X-ray beam close to normal emission (90°-α). The spectra, taken with photon energy of 515 eV, were measured in normal emission by means of a hemispherical electron analyzer with an angular acceptance angle of 2° and an overall energy resolution of 200 meV. The binding energy (BE) was calibrated by setting the BE position of Au4f7/2 peak to 84.0 eV. All spectra were corrected by subtracting a Shirley-type background. Analysis of the XPS peaks and their fitting was performed using CasaXPS software.

X-ray absorption spectra measurements were taken in partial electron yield using a channeltron detector equipped with a front grid biased at negative voltage (−370 V) to filter out the low energy secondary electrons. The NEXAFS spectra at the nitrogen and carbon K-edge were measured with the resolution set to ∼80 meV while keeping the sample at a constant grazing angle of 6°. The orientation of the surface with respect to the photon beam polarization was changed from *s*-polarization to close to *p*-polarization by rotating the sample coaxially to the photon beam axis. NEXAFS spectra were reported in the form of a normalized absorption amplitude (*I*_(E)_  =  *I*/*I*_(reference)_), using NEXAFS measured on bare Au(111) as a reference (*I*_(reference)_). Tilt angle (*θ*), relative to the surface plane, was calculated based on the following formula: 
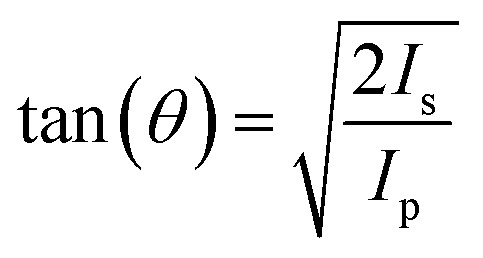
*n* which *I*_s_ and *I*_p_ represent the *s*-polarized and *p*-polarized signal amplitudes, respectively.^43^

All electronic structure calculations were performed within the framework of density functional theory, using the plane-wave-based Vienna *ab initio* simulation package (VASP)^44^ with PAW^45,46^ pseudopotentials, and the PBE^47^ exchange–correlation functional. Due to significant van der Waals interactions between the NTP molecules and the metal substrate, van der Waals dispersion corrections were calculated using the Tkatchenko–Scheffler method.^48^ We verified that the qualitative results were also consistent with calculations we performed using the non-local correlation functional, optPBE-vDW,^49–52^ which approximately accounts for dispersion interactions.

Results were converged to an accuracy of approximately 0.03 eV, in relation to the cutoff energy for the planewave basis (converged at 400 eV), the vacuum length (set at 20 Å above the metal surface), and the *k*-point mesh density. The Au(111)–NTP complex was modelled by a unit cell consisting of a Au substrate of 4 layers of 6 × 6 Au atoms in the horizontal plane and either a lone NTP molecule or evenly spaced close-packed arrays of NTP molecules. We investigated various close-packing configurations and here we present representative results of moderate close-packing with 3 molecules per unit cell in which each molecule is translated by 5.87 Å (one third of the length of the unit cell in the horizontal plane) in one direction and dense close-packing with 9 molecules per unit cell, in which each molecule is translated by 5.87 Å in 2 independent directions, forming a 3 × 3 close-packing configuration. The structures were geometrically optimized using ionic relaxation with the conjugate gradient algorithm until the convergence criteria of all forces smaller than 2 meV Å^−1^ was reached. All atoms were free to move except the bottom two Au layers, which were kept fixed with the interatomic distance determined from minimizing the energy of bulk Au. For a unit cell with *n* NTP molecules, we calculated the binding energy per unit cell of the NTP molecules by comparing the relaxed energy of the NTP molecule–Au(111) complex with the energy of the isolated Au(111) relaxed slab plus the energy of an isolated relaxed NTP molecule. The binding energy per molecule was calculated by dividing the binding energy per unit cell by *n*.

## Results and discussion

Self-assembled monolayers of *p*-NTP and *o*-NTP were prepared on Au(111) single crystal under ultra-high vacuum conditions to probe the influence of adsorption geometry on –NO_2_ reducibility. The chemical reactivity and anchoring geometry of NTPs were identified by conducting synchrotron-based XPS and NEXAFS measurements at ALOISA beamline (Elettra Synchrotron, Italy).

**Fig. 1:**
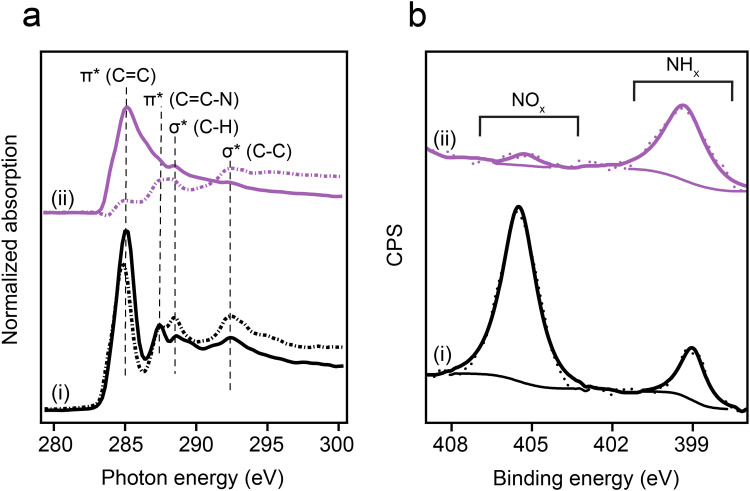
Spectroscopic characterization of *p*-NTP on Au(111). (a) Carbon k-edge NEXAFS spectra measured at *p*- and *s*-polarizations (solid and dotted lines, respectively) and (b) N1s XPS spectra. Spectroscopic measurements were conducted at room temperature (spectra i) and after exposure to 230 °C and 1000 L H_2_ (1 L = 10^−6^ torr s) (spectra ii).

The anchoring geometry of *p*-NTPs on Au(111) was analyzed by conducting carbon k-edge NEXAFS measurements at *p*- and *s*- polarizations (Fig. 1a, spectra i, *p*- and *s*-polarizations marked by solid and dotted lines, respectively). The most dominant peak was probed at 285.0 eV and correlated to C1s → π*_(C=C)_ transition (Fig. 1a, spectra i). Three minor peaks were detected at 287.4, 288.6 and 292.5 eV, correlated to C1s → π*_(C=C–N)_, C1s → σ*_(C–H)_ and C1s → σ*_(C–C)_ transitions, respectively.^53^ In the π* region (280–287.5 eV), higher amplitudes were identified for the *p*-polarized spectrum. An opposite trend was obtained in the σ* region (287.5–295 eV), in which higher amplitudes were detected in the *s*-polarized spectrum. Quantitative analysis of the difference in the C1s → π*_(C=C)_ peak amplitude for the *p*- and *s*-polarized spectra revealed that the aromatic ring is positioned at an angle of 52° with respect to the Au(111) surface (calculation details are discussed in the experimental section).

Nitro reduction yield was analyzed by conducting N1s XPS measurements. The N1s XPS spectrum of *p*-NTP/Au(111) showed two peaks (Fig. 1b, spectra i). The high energy peak (405.5 eV) was correlated to the nitro group, whereas the low energy peak (399.0 eV) was correlated to the reduced nitrogen species.^54^ The high to low energy XPS peaks area ratio was 4 : 1. This ratio indicates that 20% of the nitro groups were already reduced at room temperature, potentially due to the presence of hydrogen atoms on the Au surface following surface anchoring of thiols.^55^ Additional possible hydrogen source for the hydrogenation reaction are residual hydrogen molecules in the UHV system in which the experiments took place.^56^

**Fig. 2:**
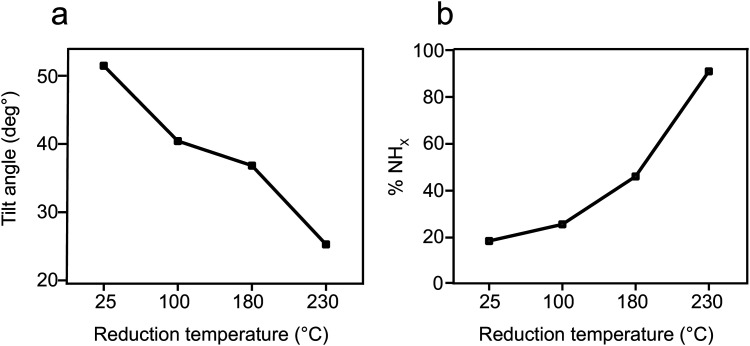
Orientation and reactivity of *p*-NTP/Au(111) as function of reduction temperature. (a) Tilt angle of the phenyl ring with respect to the metal surface, based on analysis of Carbon k-edge NEXAFS data and (b) percentage of the NH_*x*_ groups, based on analysis of N1s XPS peak area.

Exposure of the sample to reducing conditions (1000 L H_2_ at 230 °C; 1 L = 10^−6^ torr s) led to reorientation of the *p*-NTPs from an upright-tilted into a flat lying position, as identified by the positive dichroism in Carbon k-edge NEXAFS spectra (Fig. 1a, spectra ii). The reorientation was coupled with enhanced nitro reduction yield of 90%, as quantified by analysis of the NH_*x*_:NO_*x*_ peaks area ratio in the N1s XPS spectra (Fig. 1b, spectra ii). The gradual transformation in the averaged orientation of *p*-NTPs from tilted into flat-lying position as a function of the reduction temperature and the coupled increase in nitro reduction yield were analyzed based on NEXAFS and XPS data (Fig. S2, ESI†) and the results are summarized in Fig. 2a and b, respectively.

Nitrogen k-edge NEXAFS measurements showed that the –NO_2_ group of *p*-NTP was positioned in the same plane as the aromatic ring (Fig. S2, ESI†). This positioning was also detected in DFT calculations, and is energetically preferred since it enables additional resonance structures between the nitro group and the aromatic ring. Annealing of *p*-NTP/Au(111) to higher temperature than 230 °C led to chemical deformation of the SAM, as indicated from the pattern change in the S2p XPS spectra (Fig. S3, ESI†).

**Fig. 3:**
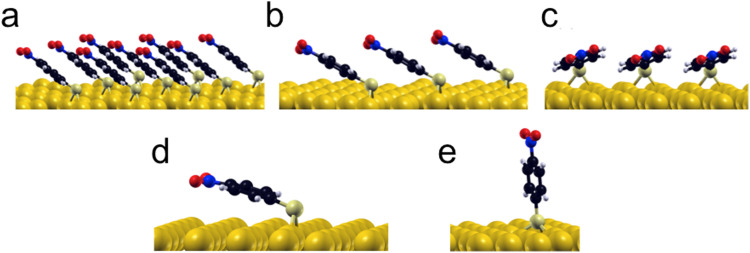
DFT calculations of the optimized adsorption geometries of *p*-NTPs on Au(111) at high packing density in which the phenyl rings are orientated parallel to each other (tilt angle = 40°, BE = 2.46 eV) (a), at intermediate packing density with the phenyl rings orientated parallel to each other (tilt angle = 33°, BE = 2.46 eV) (b) or orientated parallel to the Au surface (tilt angle = 18°, BE = 2.42 eV) (c) and at low packing density with stable flat orientation (tilt angle = 15°, BE = 2.43 eV) (d) and near-vertical metastable state (tilt angle = 82°, BE = 2.20 eV) (e).

In order to elucidate the energetic cause for *p*-NTP reorientation, the anchoring geometry and binding energies of *p*-NTP on Au(111) at various surface densities were calculated by DFT (Table S1, ESI†). At the highest packing densities, where the molecules are close to each other and have the lowest geometrical freedom (3 × 3 molecules per unit cell) the aromatic rings are aligned and interact with each other, with tilt angle of 40° and binding energy (BE) value of 2.46 eV (Fig. 3a). At lower packing density in which there are 3 molecules per unit cell, two stable orientations were observed with similar binding energies: (i) at a tilt angle of 33° with similar geometry to the high density state and with the same binding energy (BE) of 2.46 eV (Fig. 3b), and (ii) at a tilt angle of 18° and BE of 2.42 eV, where the phenyl ring is aligned and interacts with the surface as presented in Fig. 3c. At the lowest surface density, where intermolecular interactions are negligible, the flat orientation (tilt angle of 15° and BE of 2.43 eV, Fig. 3d) was found optimal. However, the high geometric freedom at low surface densities led also to a metastable near-vertical orientation (tilt angle of 82° and BE of 2.20 eV, Fig. 3e).

**Fig. 4:**
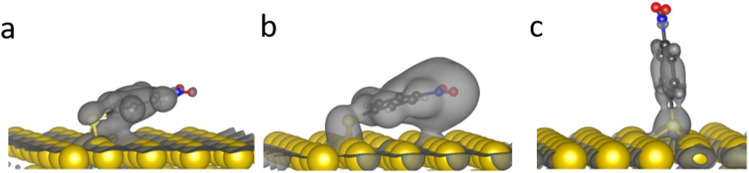
Orbital overlap between *p*-NTP and Au(111) at tilt angles of 15° (a and b) and 82° (c).

The influence of the tilt angle on surface-adsorbate interactions was probed by DFT calculations of *p*-NTP orbital overlap with the Au surface. In order to isolate the surface-adsorbate interactions the orbital overlap analysis was calculated for *p*-NTP at the lowest surface density in which intermolecular interactions are negligible. At a tilt angle of 15° there is bonding between both the π orbitals of the benzene ring and C–N bond in *p*-NTP and the underlying Au surface (Fig. 4a and b, respectively). This greater orbital overlap contributes to the increase in the binding energy and can impact the nitro reduction affinity. At the near-vertical tilt angle of 82°, only the sulfur atom had a direct interaction with the Au surface (Fig. 4c), thus rationalizing the low reactivity of molecules at this tilt angle.

Integration of the experimental data and theoretical calculations can indicate that two subpopulations are formed at room temperature, including the densely-packed configurations at 40° (Fig. 3a) and a smaller population of less dense molecules at a metastable state with a tilt angle of 82° (Fig. 3e). The combination of these two populations led to the averaged tilt angle of 52° (Fig. 2a). Despite the 0.2 eV energy difference between the two conceivable orientations for *p*-NTP at low packing density (Fig. 3d and e), it is postulated that kinetic effects played a dominant role at room-temperature and led to the experimentally-observed metastable subpopulation of molecules with a nearly upright orientation. Following annealing to 100 °C, the subpopulation of sparsely packed *p*-NTP molecules in the near vertical metastable state reoriented to the optimal flat tilt angle (Fig. 3d). The presence of the subpopulation of more flat-lying molecules lowered the averaged tilt angle to 40°.

Exposure to elevated temperature of 180 °C provided enough thermal energy for partial desorption of *p*-NTP, as identified by 35% decrease in the S2P XPS peak area (Fig. S4–S6, ESI†).^57^ The desorption of thiols from Au surfaces has been previously studied. The desorbed species was identified either as a hydrogenated thiolate (in which the hydrogen source is expected to be the residual hydrogen in the vacuum system), a thiol radical, or a disulfide.^58,59^

At lower surface density the *p*-NTP intermolecular interactions were less influential and therefore a larger fraction of the *p*-NTPs were repositioned to lower tilt angles (Fig. 3b–d), thus further lowering the overall average tilt angle. The surface density of *p*-NTPs was further decreased following annealing to 230 °C and an averaged tilt angle of 25° was experimentally detected. Interestingly, a similar transformation in the molecular orientation from tilted to flat-lying position following thermal-induced desorption was identified for benzene on Pd (111).^60^ Carbon k-edge NEXAFS spectra showed wider spectral features following exposure to reducing conditions that led to repositioning of *p*-NTPs into a flat-lying geometry (Fig. 1). This widening can be correlated to stronger interaction of *p*-NTP with the Au surface that induced orbital overlap, as identified by DFT calculations (Fig. 4).

DFT calculations of the adsorption properties of *p*-aminothiophenol (*p*-ATP), which is the *p*-NTP reduction product, identified an optimal tilt angle of 16° for lone *p*-ATP molecules on Au(111) (Fig. S7 and Table S1, ESI†). The tilt angles for *p*-ATP at all packing densities were almost identical to those calculated for *p*-NTP validating that nitro-to-amine reduction should not noticeably modify the molecular orientation. Moreover, unlike *p*-NTP, the lone surface-anchored *p*-ATP molecule has energetic advantage over the more packed molecules (Table S1, ESI†), which can further rationalize the decrease in the overall tilt angle. The results thus far show that surface proximity of the nitro group and parallel orientation with respect to the Au surface are essential for effective nitro reduction in *p*-NTPs on Au(111).

In order to identify if the link between surface proximity, adsorption geometry and nitro reducibility can be generalized, we have analyzed the orientation-reactivity correlations in *o*-NTPs on Au(111). The nitro group in *o*-NTP is positioned in closer proximity to the metal surface than in *p*-NTP. Therefore, if surface proximity is a limiting step in the reduction process, it is expected that nitro reduction in *o*-NTPs will be facilitated under milder reaction conditions comparing to those observed for *p*-NTP.

**Fig. 5:**
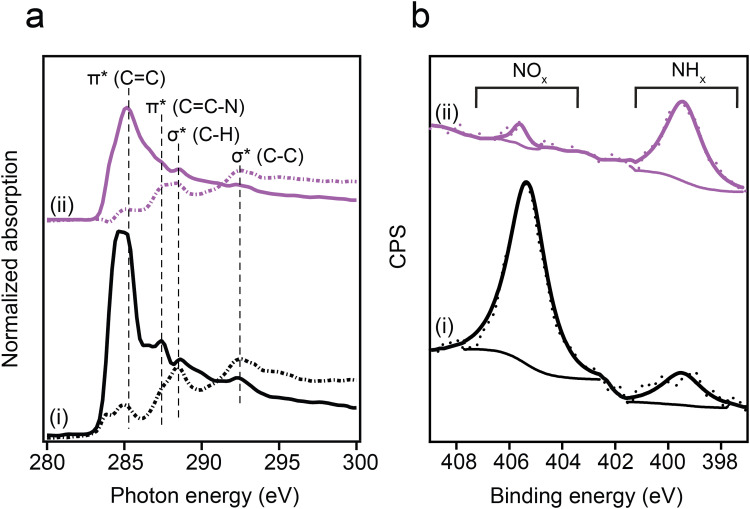
Spectroscopic characterization of *o*-NTP on Au(111). (a) Carbon k-edge NEXAFS spectra measured at *p*- and *s*-polarizations (solid and dotted lines, respectively) and (b) N1s XPS spectra. Spectroscopic measurements were conducted at room temperature (spectra i) and after exposure to 180 °C and 1000 L H_2_ (1 L = 10^−6^ torr s) (spectra ii).

Carbon k-edge NEXAFS spectra of surface-anchored *o*-NTP (Fig. 5a, spectra i) showed a significant positive dichroism. Such dichroism was detected in *p*-NTPs only after annealing to 230 °C. Quantitative analysis of the *p*- and *s*-polarized C1s → π*_(C=C)_ peaks ratio revealed that *o*-NTPs were oriented at a tilt angle of 28° with respect to the Au surface. N1s XPS spectra of *o*-NTP showed a high- to low-energy peaks area ratio of 4 : 1, indicating that 20% of the nitro groups were reduced at room temperature (Fig. 5b, spectra i). Thus, although the distance between the –NO_2_ group of *o*-NTP and the Au surface was 3.0 ± 0.3 Å, compared to 6.6 ± 0.3 Å in *p*-NTP, the reactivity towards nitro reduction at room temperature was similar to the one detected for *p*-NTPs.

Exposure to reducing conditions (180 °C and 1000 L H_2_) did not significantly modify the anchoring geometry of *o*-NTPs, as revealed by the dichroism pattern in Carbon k-edge NEXAFS spectra (Fig. 5a, spectra ii). However, the reducing conditions led to 90% yield in nitro reduction (Fig. 5b, spectra ii). These results indicate that physical proximity was not sufficient for nitro reduction in *o*-NTP and that an elevated temperature was essential in order to overcome an activation energy barrier that prevented the initiation of the reduction process.

**Fig. 6:**
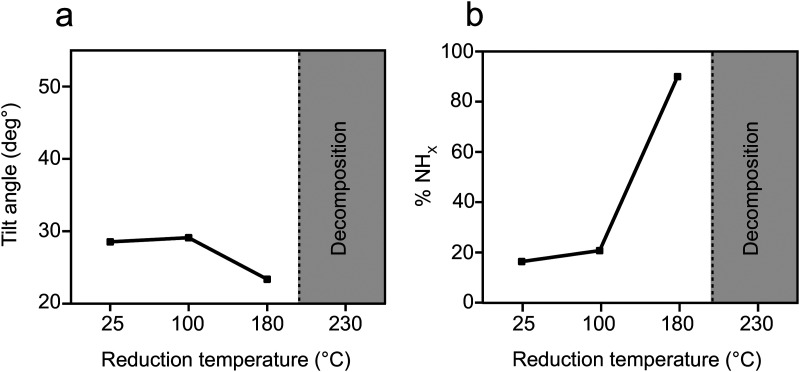
Orientation and reactivity of *o*-NTP/Au(111) as function of reduction temperature. (a) Tilt angle of the phenyl ring with respect to the metal surface, based on analysis of Carbon k-edge NEXAFS data. (b) Percentage of the NH_*x*_ groups, based on analysis of N1s XPS peak area.

Quantitative analysis of the tilt angle and reactivity of *o*-NTPs on Au(111) as a function of the reduction temperature were calculated based on NEXAFS and XPS data (Fig. S8, ESI†) and the results are shown in Fig. 6a and b, respectively. This analysis reveals that *o*-NTPs were adsorbed in a flat-lying orientation and their tilt angle was moderately changed from 28° to 23° following exposure to reducing conditions. The distance between the nitrogen atom in *o*-NTP and the Au surface was practically constant (modified from 3.0 ± 0.4 to 2.9 ± 0.4 Å) as the tilt angle decreased from 28° to 23°. Thus, the enhanced reactivity at 180 °C cannot be associated with changes in the adsorption geometry of *o*-NTP but can be correlated to the energy required for nitro group activation. It should be noted that *o*-NTPs were characterized with deteriorated thermal stability in comparison to *p*-NTPs and decomposition of *o*-NTPs already took place at *T* > 180 °C.

The asymmetric molecular structure of *o*-NTP prevented the formation of a highly packed monolayer and the surface density of *o*-NTPs was lower by ∼40% than that of *p*-NTPs (Fig. S4–S6, ESI†). The preference towards lower surface density of *o*-NTP was identified as well in DFT calculations where the binding energy per molecule for the most densely packed configuration was lower by 0.4–0.45 eV than the less dense configurations (Table S1, ESI†). At low packing densities, DFT calculations show that the *o*-NTP molecules are more stable in the flatter orientations (tilt angles of 20° for lone molecules and 31° for intermediately packed molecules, see Table S1, ESI†), which led to the preferred flat lying position of *o*-NTPs on the Au surface. It should be noted that for the reduced molecule, *o*-ATP, the most stable geometry was for the lone molecule at a tilt angle of 21°, corresponding to the experimental observation.

**Fig. 7:**
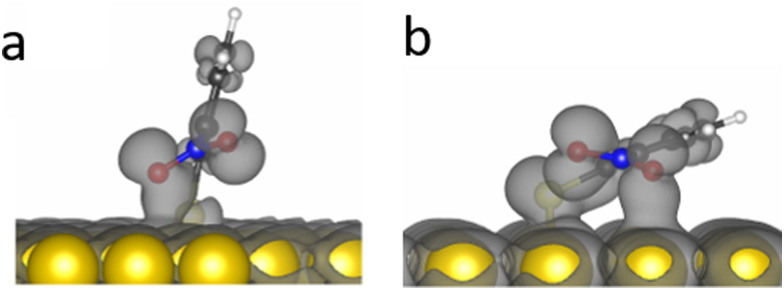
Orbital overlap of *o*-NTP on Au(111) at tilt angle of 76° and 20° (a and b, respectively).

DFT calculations demonstrated that the nitro group in *o*-NTP rotates away from the plane of the aromatic ring in order to be in a close to parallel position to the Au surface and clear bonding was observed between the oxygen atoms and the Au surface even at a tilt angle of 76° (Fig. 7a). This interaction was more noticeable at a tilt angle of 20° (Fig. 7b), in which both higher surface proximity and parallel nitro position were identified and led to an optimal position of the *o*-NTP molecule (Table S1, ESI†). Such parallel orientation of the nitro group was also detected in NEXAFS data (Fig. S8, ESI†).

The improved orbital overlap between the nitro group of *o*-NTP and the Au surface, in comparison to the one detected for *p*-NTP, is the result of higher surface proximity and can lead to weaker N–O bonds, thus rationalizing the enhanced reactivity toward nitro reduction in *o*-NTP. The bonding between the nitro group of *o*-NTP and the Au(111) surface involves the p-orbitals of the oxygen atom. Unlike *o*-NTP where the nitro group rotates away from the plane of the aromatic ring to be parallel to the Au surface, in *p*-NTPs the nitro group was positioned in the same plane as the aromatic ring. Thus, even at a tilt angle of 15°, the oxygen atoms in *p*-NTP are positioned further from the surface than in *o*-NTP and only the π orbitals of the benzene ring and C–N bond interact with the Au surface, with almost no interaction of the oxygen p orbitals (Fig. 4).

**Scheme 1:**
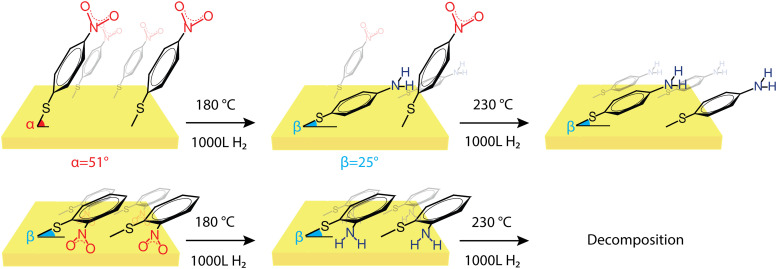
Schematic representation of the orientation and reactivity of *p*-NTP and *o*-NTP on Au(111). *p*-NTP (upper panel) is adsorbed at an upright-tilted angle and upon annealing it adopts a flat-lying orientation, followed by a reduction of the nitro group. *o*-NTP (lower panel) is adsorbed in a flat-lying orientation, and the nitro group is reduced upon exposure to milder conditions compared to *p*-NTP, followed by a decomposition of the molecule.

The insights about the influence of surface proximity on nitro reduction affinity for *p*-NTP and *o*-NTP on Au(111) are depicted in Scheme 1. At mild temperature (*T* = 25-100 °C) the dominant population of *p*-NTPs was anchored on the Au surface at an upright-tilted position and high packing density (Scheme 1, top panel). Exposure to reducing conditions and elevated temperature (*T* = 180 °C) led to partial desorption that lowered the surface density of *p*-NTPs and enabled their reorientation into a closer to flat-lying position. A correlation was identified between the percentage of *p*-NTPs that accumulated a flat-lying position and the nitro reduction yield. The parallel positioning of the benzene ring and nitro group with respect to the Au surface led to orbital overlap between the π bonds of the C–N and benzene ring in the flat-lying *p*-NTP and the Au surface (Fig. 4). It is postulated that these interactions enhanced the affinity toward nitro reduction, reaching a close to full conversion at 230 °C. Unlike *p*-NTPs, Au-anchored *o*-NTPs accumulated a close to flat-lying position (tilt angle = 28°) already at room temperature (Scheme 1, bottom panel). Although nitro groups in *o*-NTPs were positioned in high proximity to the Au surface, nitro reduction was facilitated only once reduction temperature reached 180 °C.

It should be noted that for both *p*-NTP and *o*-NTP, nitro reduction occurred when the nitro groups were approximately parallel to the surface and in close proximity to it (distance of nitro group in *o*- and *p*-NTP from the Au surface was 2.9 ± 0.4 and 4.5 ± 0.3 Å, respectively). The parallel positioning of the nitro group led in *o*-NTP to an interaction of the oxygen atoms with the Au surface (Fig. 7), while in *p*-NTP the C–N and benzene π bonds interacted with the Au surface and negligible interaction was obtained between the Au surface and the oxygen atoms (Fig. 4). It is hypothesized that the different level of surface interaction in *o*- and *p*-NTP influenced their affinity toward nitro reduction. However, for both molecules a close to parallel position was essential for facilitating the reduction process. Interestingly, a similar influence of the positioning on reactivity pattern was identified for C=O hydrogenation in ketones, in which parallel adsorption of the carbonyl groups was essential for its activation.^26,30–32^

It is important to note that the use of tethered nitro-benzene molecules was essential in order to observe the influence of various geometries on the reactivity. In the absence of an anchoring group, the nitrobenzene molecules adopt a flat lying configuration due to strong interaction between the benzene ring and the metal surface. The preference of this configuration will hinder the decoupling of geometry and reactivity.^34,35^ It was shown that the addition of side groups can modify the adsorption geometry and therefore, in a limited way, impact the reactivity.^35^

The mechanistic insights that stem from the above-described results indicate that selective reduction of nitro groups in multifunctional molecules can be tuned by directing the adsorption geometry of the reactant molecules on catalytic surfaces. The essential precondition of parallel adsorption configuration of the nitro group introduces new strategies to induce or hinder selective nitro reduction. These strategies include altering the molecular structure of the reactant with substituents of different steric demand. For example, bulky steric groups can be positioned on different molecular sites in a remote or adjacent position to the nitro group in order to facilitate or hinder, respectively, nitro reduction. The adsorption geometry and hence the affinity toward nitro reduction can be also potentially tuned by altering the local adsorption-site environment. This can be achieved by the choice of the solvent, which was demonstrated to compel the adsorbed molecules to align with certain adsorption orientations.^61–64^ Alternatively, co-adsorbates can be used to direct the adsorption geometry of adsorbate molecules on the catalytic surface.^62,65^ These approaches for tuning the adsorption geometry of nitro-aromatics can be utilized for diminishing the tendency toward a flat-lying adsorption geometry and thus moderate nitro reduction efficiency.

## Conclusions

The dominant impact of adsorption geometry on the affinity toward nitro reduction in nitro-aromatics was identified by using well-defined model systems constructed of *p*-NTP and *o*-NTP monolayers on Au(111) and their analysis by polarized NEXAFS measurements and DFT calculations. Nitro reduction in *p*-NTP was facilitated once the molecule was oriented in a close to flat-lying position and approximately parallel to the surface. This position enabled interaction of the C–N and benzene π-bonds with the Au surface, as identified by DFT calculations. *o*-NTP was positioned in a close to flat-lying orientation on Au(111) already at room temperature and the nitro group rotated away from the plane of the benzene ring to be in an even closer to parallel position to the surface. The higher surface proximity of the nitro group in *o*-NTP led to interaction of the oxygen p-orbital with the Au surface. This positioning led to a high reduction yield at a milder temperature (180 °C) than the one required for nitro reduction in *p*-NTP (230 °C). Nitro reduction was therefore facilitated once strong molecular interactions were formed with the Au surface and such interactions require that the nitro group will be positioned in close proximity and approximately parallel to the surface. Hence, it is deduced that selective nitro reduction in multifunctional reactants can be obtained by modifying the adsorption geometry of the reactant molecule, which can be achieved by using multifunctional reactant molecules or by altering the geometry of the catalytic surface.

## Conflicts of interest

There are no conflicts to declare.

## Supplementary Material

CP-024-D2CP02832H-s001
