# Supplementary material for: The influence of adsorption geometry on the reduction affinity of nitroaromatics on Au(111)
Source: Phys Chem Chem Phys. 2022 Sep 12;24(37):22960–70. doi: 10.1039/d2cp02832h (PMC9756653; doi:10.1039/d2cp02832h)
Supplement: CP-024-D2CP02832H-s001 [file CP-024-D2CP02832H-s001.pdf]

Electronic Supplementary Material (ESI) for Physical Chemistry Chemical Physics.
This journal is © the Owner Societies 2022

Supporting Information

The Influence of Adsorption Geometry on the Reduction Affinity of Nitroaromatics on Au (111)

Iris Berg[a,b +], Helen Eisenberg[a,c +], Shahar Dery[a,b +], Tehila Shahar[a,b], Albano Cossaro[d], Alberto Verdini[d], Luca Floreano[d], Tamar Stein[a,c *] and Elad Gross[a,b *]

[a] Institute of Chemistry, The Hebrew University, Jerusalem 91904, Israel

[b] The Center for Nanoscience and Nanotechnology, The Hebrew University, Jerusalem 91904, Israel

[c] The Fritz Haber Center for Molecular Dynamics Research, The Hebrew University, Jerusalem 91904, Israel

[d] CNR-IOM, Laboratorio Nazionale TASC, Basovizza SS-14, Trieste 34012, Italy

[+] These authors equally contributed to this work

[*] Corresponding authors' email address: tamar.stein@mail.huji.ac.il, elad.gross@mail.huji.ac.il

Table S1: Calculated tilt angles between the aromatic ring plane and the horizontal Au (111) surface and corresponding binding energies. The binding energies and molecular geometries (tilt angles) are given for geometrically relaxed stable states. The effect of close-packing on the molecular binding energy and tilt angle was investigated. The following systems were calculated: Lone molecules, namely one molecule per unit cell. Close-packing with 3 molecules per unit cell in which each molecule is translated by 5.87 Å (one third of the length of the unit cell in the horizontal plane) in one direction. Very close packing with 9 molecules per unit cell, in which each molecule is translated by 5.87 Å in 2 independent directions, forming a 3x3 close-packing configuration.

Molecule	Packing	Angle (°)	Binding Energy (eV)
p-NTP	3x3 molecules per unit cell	40	2.46
	3 molecules per unit cell	33	2.46
	3 molecules per unit cell	18	2.42
	Lone molecule	82	2.20
	Lone molecule	15	2.43
p-ATP	3x3 molecules per unit cell	38	2.19
	3 molecules per unit cell	32	2.21
	3 molecules per unit cell	18	2.20
	Lone molecule	85	2.07
	Lone molecule	16	2.26
o-NTP	3x3 molecules per unit cell	71	1.70
	3 molecules per unit cell	38	2.11
	3 molecules per unit cell	31	2.15
	Lone molecule	76	2.02
	Lone molecule	20	2.14
o-ATP	3x3 molecules per unit cell	61	1.89
	3 molecules per unit cell	43	2.08
	3 molecules per unit cell	27	2.19
	Lone molecule	21	2.30

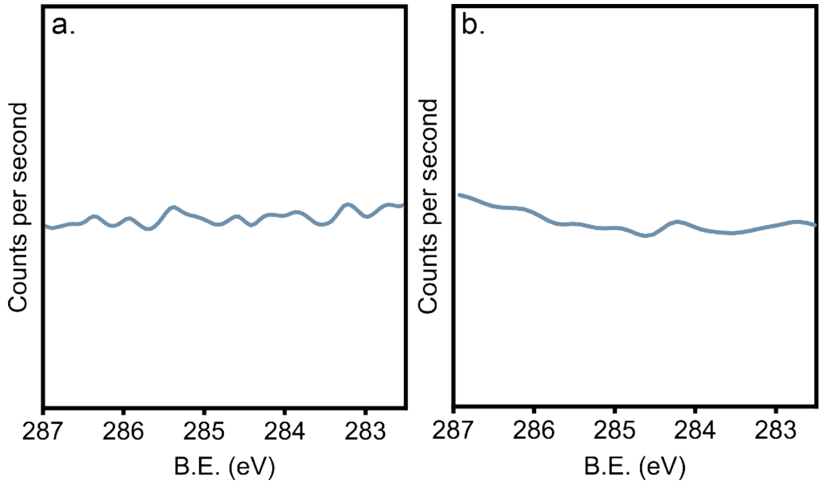

Figure S1: C1s XPS spectra of (a) Au (111) and (b) Pt (111) after three cycles of sputter and annealing.

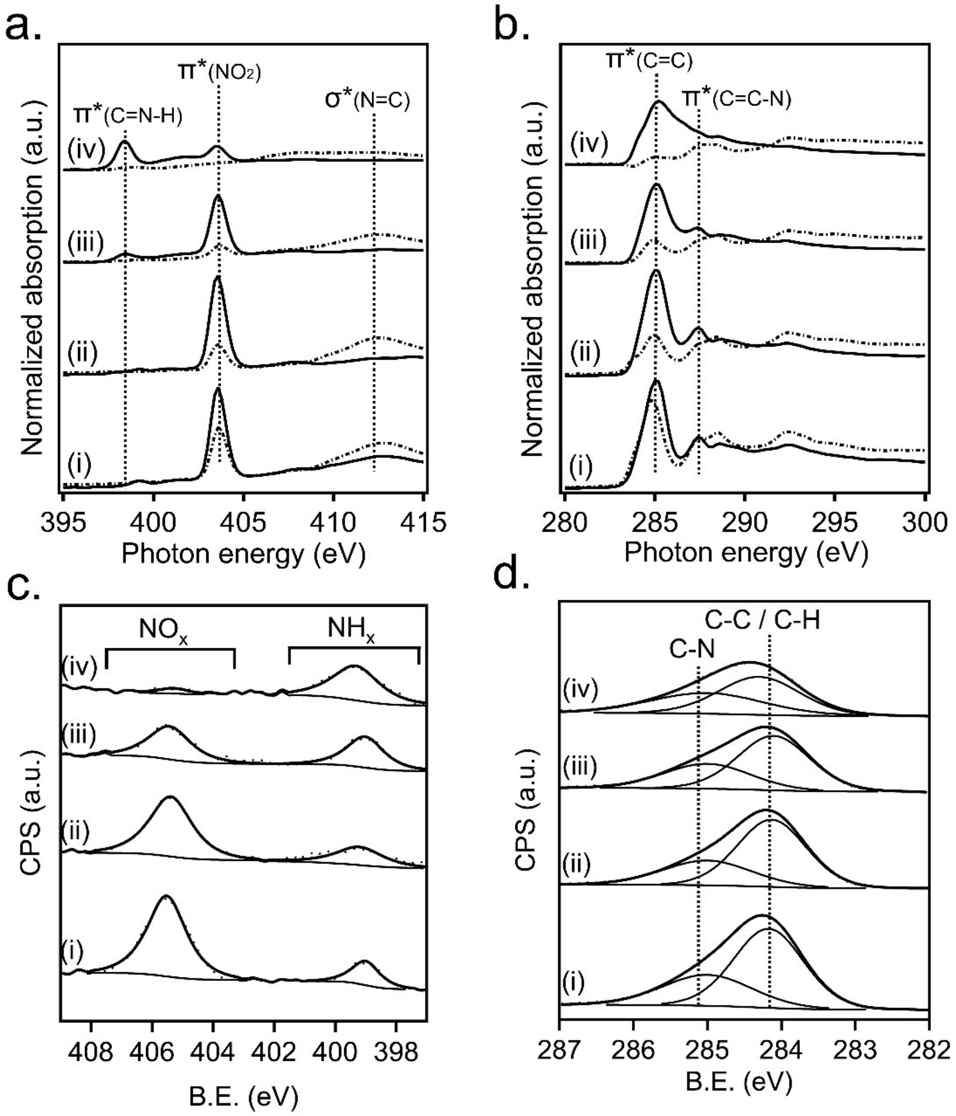

Figure S2: Spectroscopic data of p-NTP on Au (111): (a) Nitrogen k-edge and (b) Carbon k-edge NEXAFS spectra were measured at room temperature (spectra i), and after annealing to 100 ºC (spectra ii), 180 ºC (spectra iii) and 230 ºC (spectra iv). All elevated temperatures were accompanied by 1000L H_2. P- and s-polarized NEXAFS spectra were marked by solid and dotted lines, respectively. (c) N1s and (d) C1s XPS spectra. Gaussian fittings were added to the C1s XPS signal

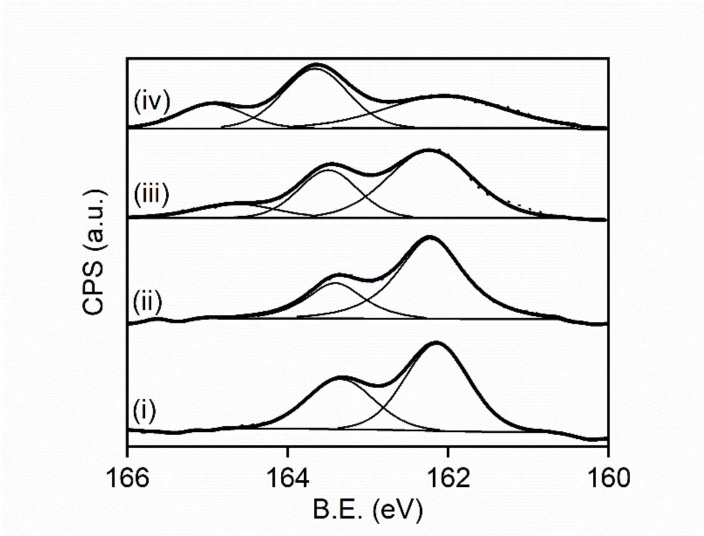

Figure S3: S2p XPS spectra of p-NTP on Au (111) measured at room temperature (spectrum i), and after annealing to 100 ºC (spectrum ii), 180 ºC (spectrum iii) and 230 ºC (spectrum iv). Gaussian fittings were added to the XPS spectra.

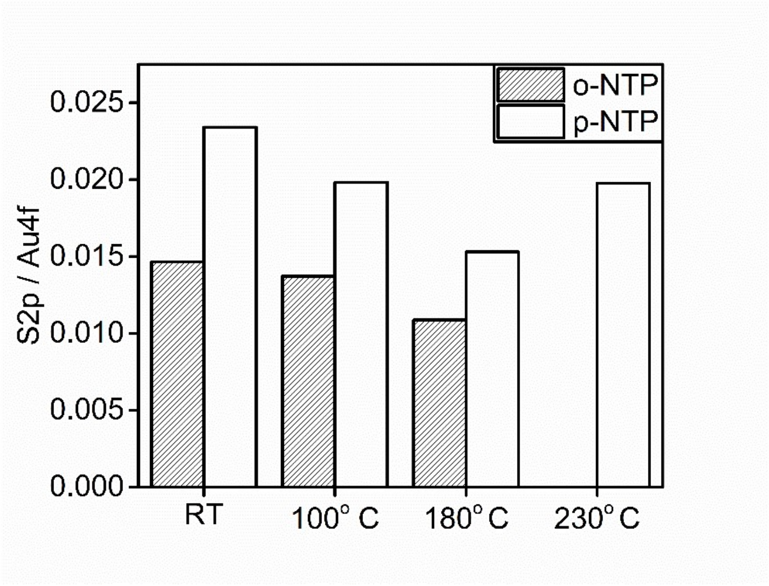

Figure S4: S2p / Au4f XPS peaks area ratio of o-NTP and p-NTP on Au (111). Annealing to elevated temperatures was accompanied with exposure to 1000L H_2.

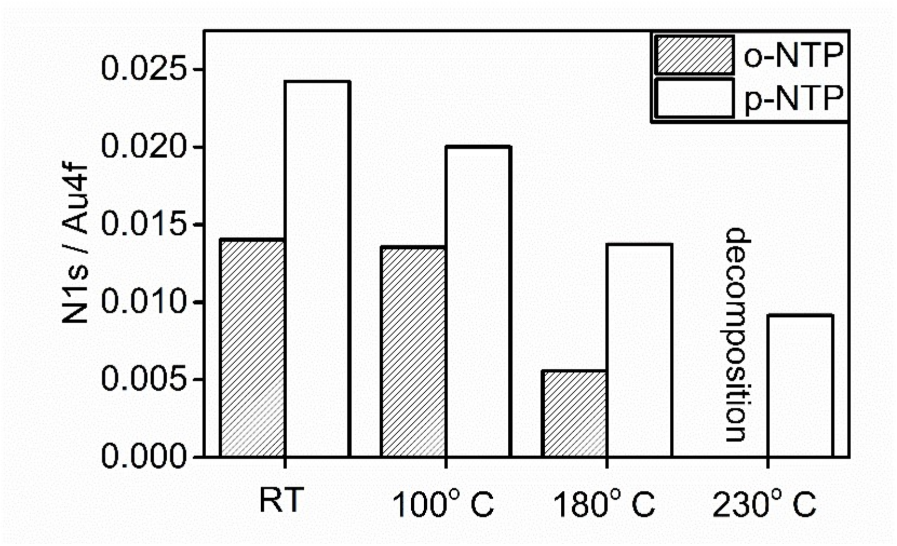

Figure S5: N1s / Au4f XPS peaks area ratio of o-NTP and p-NTP on Au (111). Annealing to elevated temperatures was accompanied with exposure to 1000L H_2.

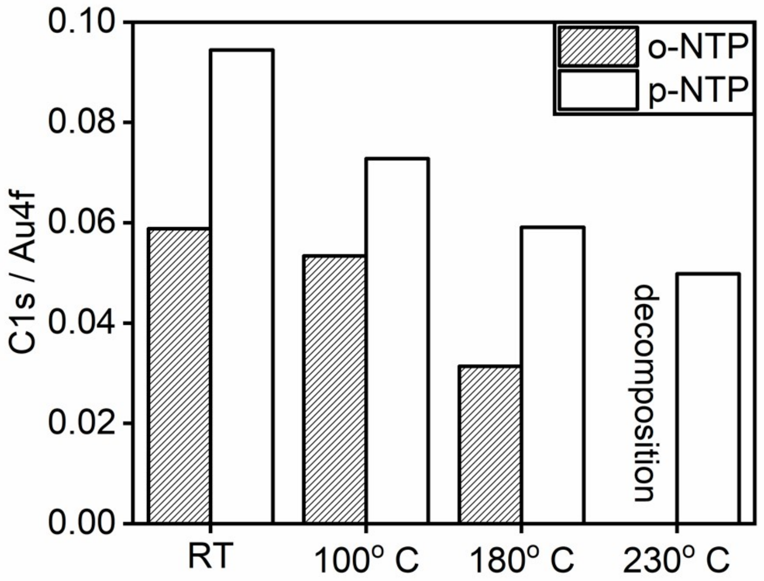

Figure S6: C1s / Au4f XPS peaks area ratio of o-NTP and p-NTP on Au (111). Annealing to elevated temperatures was accompanied with exposure to 1000L H_2.

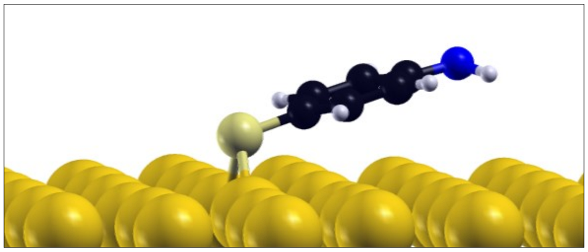

Figure S7: DFT calculations of the adsorption geometries of p-ATP on Au (111).

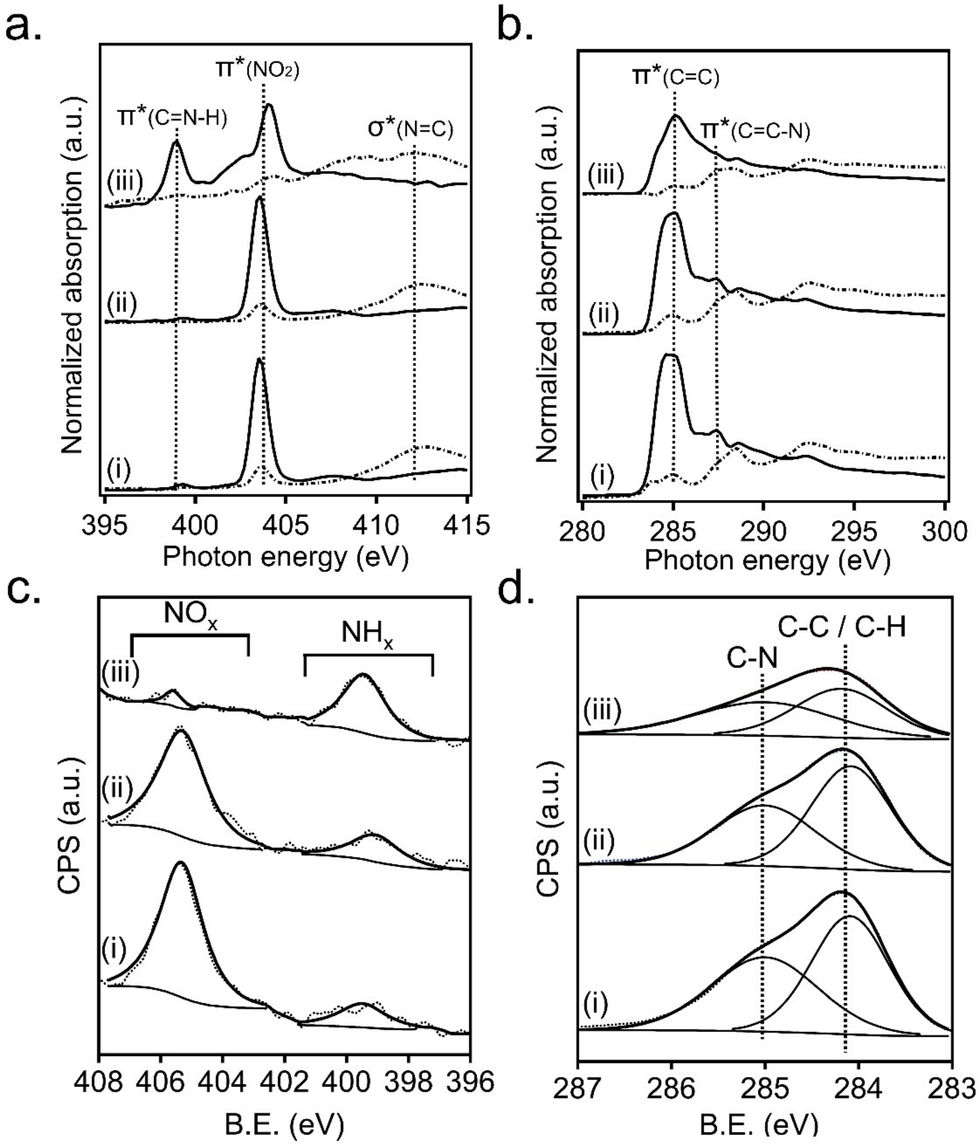

Figure S8: Spectroscopic data of o-NTP on Au (111): (a) Nitrogen k-edge and (b) Carbon k-edge NEXAFS spectra were measured at room temperature (spectra i), and after annealing to 100 ºC (spectra ii) and 180 ºC (spectra iii). All elevated temperatures were accompanied by 1000L H_2. P- and s-polarized NEXAFS spectra were marked by solid and dotted lines, respectively. (c) N1s and (d) C1s XPS spectra. Gaussian fittings were added to the C1s XPS signal
